# A Systematic Review of the Psychosocial Mechanism Underlying the Pathway from Intra-Familial Victimization in Childhood to Intimate Relationship Violence in Adolescence and Adulthood

**DOI:** 10.1177/15248380251320979

**Published:** 2025-03-04

**Authors:** Natnicha Boonyananth, Lorraine Swords

**Affiliations:** 1 Trinity College Dublin, Ireland

**Keywords:** childhood victimization, child maltreatment, intimate partner violence, adolescent dating violence, attachment, early maladaptive schema, sense-of-self

## Abstract

Intra-familial childhood victimization (IF-CV) has been associated with increased involvement in relationship violence in adolescence and adulthood. Drawing from developmental theories by Bowlby on attachment styles, Young on Early Maladaptive Schema (EMS), and Erikson on psychosocial development (i.e., Sense-of-Self), we propose a conceptual model to explain this mechanism. A Mixed-Methods Systematic Review was conducted to synthesize evidence in support of this hypothesis. Three databases (*PsycINFO*, *PubMed*, and *Web of Science*) were searched, with additional manual searching using Google Scholar and reference tracing. A total of 17 peer-reviewed studies met the inclusion criteria. Results indicated that attachment styles may moderate, while EMS mediate, the link between IF-CV and relationship violence. These findings suggest that attachment may function as a primary protective factor against the cycle of violence, through its effect on EMS. Aspects of Sense-of-Self, such as self-control, self-blame, self-worth, and self-esteem, also emerged as strong contributors to the mechanism. However, none of the studies have investigated identity resolution as a potential mediator of the pathway. Neither have the three factors (attachment, EMS, and Sense-of-Self) been studied previously in relation to one another. The effect of gender is inconclusive, while the impact of age has not been examined in any of the papers. Future research should address these gaps to provide further insights into the mechanism and better inform interventions.

Felitti et al. (1998) presented a landmark study that revealed a significant link between adults’ history of childhood adversity and their current health outcomes. The investigated factors included child maltreatment (psychological, physical, and/or sexual abuse, and neglect), witnessing of domestic violence at home, and household dysfunctions (e.g., parental incarceration). Since then, a multitude of research has produced results that substantiated the original findings (Petruccelli et al., 2019; Scully et al., 2019) and expanded the scope of childhood adversity to include extra-familial factors, such as social and economic hardship (Alhowaymel et al., 2021). In this systematic review, only literature focusing on intra-familial childhood victimization (IF-CV, including child maltreatment and witnessing of domestic violence) was investigated to gain deeper insights into the effects of this specific type of childhood adversity on later outcomes.

Researchers have identified increased risks for IF-CV survivors to repeat the pattern of violence within intimate relationships. For example, young people who witnessed violence at home are more likely to engage in adolescent dating violence (Forke et al., 2018). Adults with IF-CV experiences are also more likely to perpetrate relationship violence (Kimber et al., 2018; Li et al., 2020) and become victimized (Fanslow et al., 2021).

Despite the established links between IF-CV and relationship violence, little is known about the mechanism through which it may operate. Several researchers have turned to developmental theories for conceptual explanations. Among the most influential is attachment theory (Bowlby, 1978), which suggests that children’s early interactions with caregivers create the blueprint for future social relations. Individuals’ attachment styles are classified as either secure or insecure (anxious/preoccupied, avoidant/dismissive, or disorganized/fearful; Ainsworth, 1989). Secure attachment is formed when caregivers are sensitive and responsive to children’s needs, helping them learn to emotionally regulate in times of distress. In the case of IF-CV, abusive parents present a source of threats, to which children may respond with high anxiety and become preoccupied with seeking connections with their unavailable caregivers. Alternatively, they may dismiss their caregivers to avoid potential physical or emotional harm. Some children may respond with both high anxiety and avoidance, creating disorganized patterns of behaviors also characterized as fearful.

Although attachments formed during early childhood can leave a crucial imprint on one’s development, it is important to note that attachment forming is a lifelong process (Howe, 2011). Hence, IF-CV occurring throughout different stages of childhood bears the potential to greatly disrupt one’s development. Studies show that adults tend to carry over their attachment styles formed during childhood to their current intimate relationships, which could impact their patterns of responses in times of conflict (Spencer et al., 2021). For example, securely attached individuals are able to regulate their emotions and successfully resolve conflicts while maintaining closeness with their partners. In contrast, anxiously attached individuals may resort to violence to keep their partners from leaving, while those with an avoidant attachment may use it as a strategy to push them away (Allison et al., 2008).

In addition to emotion regulation, attachment styles may also affect relationship quality through cognitive models of self and others, that is, “Internal Working Models” (IWMs; Bretherton, 1992). Securely attached individuals tend to see themselves as worthy and others as a reliable source of support. In contrast, insecurely attached individuals may see themselves as incompetent and undeserving, and others as untrustworthy. Negative IWMs have been theorized to bias individuals’ perceptions of others’ behaviors, which may lead to relational aggression through the activation of the threat response system (Riggs, 2010).

The concept of IWMs was further expanded by Young et al. (2003), who introduced Early Maladaptive Schemas (EMS) in Schema Therapy. Conceptualized as a dysfunctional pattern of thoughts and emotions regarding oneself and the world, 18 EMS have been identified and categorized into five domains based on unmet childhood needs (see Table 1). A recent meta-analysis has demonstrated significant associations between insecure attachment styles and EMS (Karantzas et al., 2022), substantiating the hypothesis that they may represent a subset of negative IWMs (Simard et al., 2011). According to Young et al., when EMS are activated during conflicts, individuals may maladaptively respond by surrendering, avoiding, or overcompensating. These strategies may show up in relationships as accepting abuse, avoiding closeness, or using aggression to retaliate. Consistently, studies have linked EMS with IF-CV and relationship violence involvement (Hassija et al., 2018; Pilkington et al., 2020).

**Table 1.** EMS Organized into Five Domains.

Domain	EMS
Disconnection and rejection	1. Abandonment 2. Mistrust/Abuse 3. Emotional deprivation 4. Defectiveness/Shame 5. Social isolation
Impaired autonomy and performance	6. Dependence/Incompetence 7. Vulnerability to harm/illness 8. Enmeshment/Undeveloped self failure
Impaired limits	10. Entitlement/Grandiosity 11. Insufficient self-control/self-discipline
Other-directedness	12. Subjugation 13. Self-sacrifice 14. Approval/recognition-seeking
Over-vigilance and inhibition	15. Negativity/Pessimism 16. Emotional inhibition 17. Unrelenting standards 18. Punitiveness

*Note*. EMS = early maladaptive schemas.

Both attachment and EMS theories provide valuable insights into the pathway from IF-CV to relationship violence. However, they focus on early experiences, leaving much to be explored during the developmental period before the emergence of intimate relationships in adolescence and early adulthood. Drawing from the lifespan development perspective of Erikson (1950), individuals progress through life in consecutive stages with specific psychosocial tasks to accomplish (see Table 2). While intimacy is seen as the primary task for early adulthood, it cannot be fully obtained without the successful resolution of tasks in preceding stages (i.e., identity formation in adolescence, industry in school age, initiative in preschool, autonomy in early childhood, and trust in infancy). Consistent with attachment theory, Erikson viewed the early development of trust and autonomy as the foundation for lifelong development. His unique contribution, however, lies in the emphasis on identity formation in adolescence. It has been theorized that unresolved identity crises may increase the likelihood of relationship violence, as individuals may become overly embedded with their partners and struggle to express their own needs (Exner-Cortens, 2014). Studies have also associated different facets of self, such as self-control, self-esteem, and self-worth to both relationship violence (Bolívar-Suárez et al., 2022; Meldrum et al., 2020; Novak & Furman, 2016) and IF-CV (Goodman et al., 2021; Jones et al., 2021). It can therefore be argued that IF-CV may lead to the disruption in forming an integrated Sense-of-Self, which may increase individuals’ susceptibility to relationship violence.

**Table 2.** Erikson’s Psychosocial Stages of Development.

Developmental Stages	Developmental Tasks
Infancy (1–2 years)	Trust vs. Mistrust
Early childhood (2–4 years)	Autonomy vs. Shame and doubt
Preschool age (4–5 years)	Initiative vs. Guilt
School age (5–12 years)	Industry vs. Inferiority
Adolescence (13–19 years)	Identity vs. Role confusion
Emerging adulthood (20–40 years)	Intimacy vs. Isolation
Adulthood (40–65 years)	Generativity vs. Stagnation
Maturity (65+ years)	Ego integrity vs. Despair

## Theoretical Integration and Present Study

**Figure 1.**
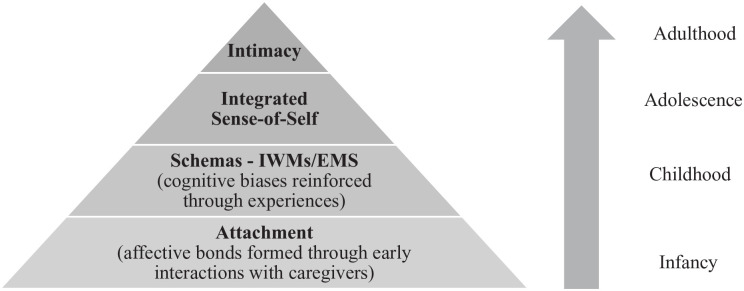
The theoretical model of the relationships between attachment, schemas, sense-of-self, and intimacy. *Note*. IF-CV = intra-familial childhood victimization.

**Figure 2.**
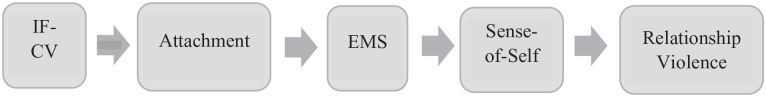
The conceptual framework of the psychosocial mechanism underlying the IF-CV to relationship violence pathway. *Note*. IF-CV = intra-familial childhood victimization.

The conceptual integration of attachment, EMS, and Erikson’s psychosocial development theories is illustrated in Figure 1. This study aims to apply this framework to explore the pathway from IF-CV to relationship violence in adolescence and adulthood (see Figure 2). Although several studies have investigated the mechanism through the lens of attachment, EMS, and/or Sense-of-Self, findings have not been formally synthesized under an integrated framework. Therefore, we conducted a Mixed-Methods Systematic Review (MMSR) to consolidate existing literature and find evidence for our theoretical conceptualization. By gaining deeper insights into this mechanism, the research has the potential to identify areas for intervention to disrupt the cycle of violence from IF-CV to relationship violence.

## Methods

### Search Strategy

The Population, Interest, Context approach (PICo; Stern et al., 2014) was used to create appropriate search terms (see Table 3). Abstracts were searched across three databases (*PsycINFO*, *PubMed*, and *Web of Science*). Manual searching was also conducted using *Google Scholar* and reference tracing.

**Table 3.** Complete List of Search Terms.

PICo	Search Terms
**P**opulation	• (child* OR adol* OR teen* OR youth* OR “young people” OR “young person*”)
Phenomenon of **I**nterest	• (“adverse childhood experience*” OR abus* OR neglect* OR maltreat* OR “domestic violence” OR “intimate partner violence” OR “interpersonal violence” OR “interparental violence” OR “family dysfunction*” OR “dysfunctional famil*”) • (“intergenerational transmission” OR “cycle of violence” OR perpetrat* OR revictimi*ation OR victimi*ation OR “dating violence”)
**Co**ntext	• (self OR identity OR trust OR attach* OR “mother-child relationship*” OR “father-child relationship*” OR “parent-child relationship*” OR agency OR agent* OR shame OR doubt OR guilt)

### Study Selection

Studies dating from the beginning of literature to the point of searching (March 22, 2021) were included. Non-English studies were excluded as the authorship team was English-speaking. Further inclusion and exclusion criteria are outlined in Table 4.

**Table 4.** Inclusion and Exclusion Criteria.

Inclusion	Exclusion
• Peer-reviewed • English • Perpetration/Victimization of intimate relationship violence either in adolescence or adulthood • Identified factors as contributing to the pathway from IF-CV to intimate relationship violence (i.e., mediators and/or moderators in quantitative studies; explained as such in qualitative studies)	• Non peer-reviewed • Non-English • Other types of interpersonal violence (e.g., bullying) and/or household dysfunctions (e.g., divorce) • Identified factors as mere correlators of IF-CV and/or intimate relationship violence

*Note*. IF-CV = intra-familial childhood victimization.

### Search Results

**Figure 3.**
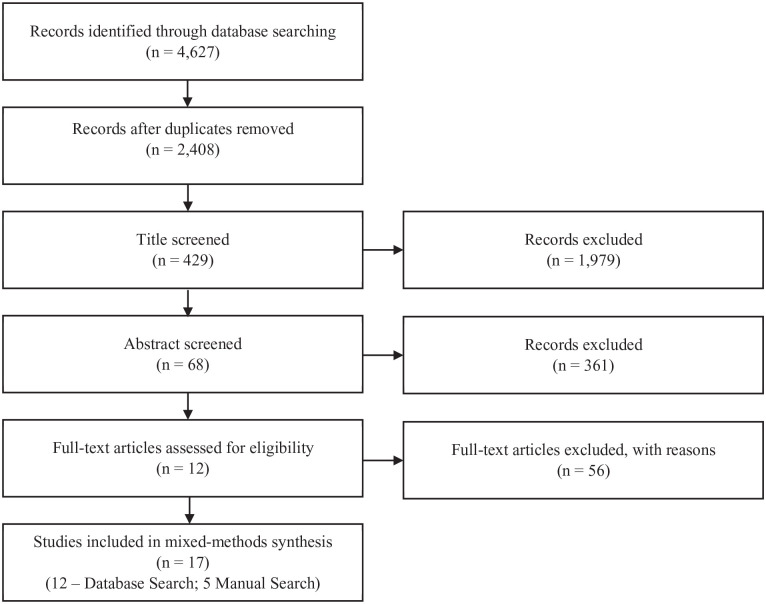
Prisma diagram.

The Preferred Reporting Items for Systematic Reviews and Meta-Analyses statement guidelines (PRISMA; Liberati et al., 2009) were used to identify and screen search results (see Figure 3). The final selection comprises 17 studies (12 from database search and five from manual search).

### Quality Assessment

Two researchers went through the search results independently to ensure satisfactory inter-rater reliability. The Mixed-Methods Appraisal Tool (MMAT; Hong et al., 2018) was also used to assess the quality of each study included.

### Data Extraction and Synthesis

The JBI methodology for MMSR (Lizarondo et al., 2019) was used to synthesize the data. Results from quantitative studies were qualified and integrated with findings from qualitative studies. They were categorized based on the psychosocial factors examined and presented together using the convergent integrated approach.

## Results

The final 17 studies have been organized based on the psychosocial factors identified. Since the study by Gay et al. (2013) examined more than one factor (attachment and EMS), their results are presented in both sections.

### Attachment

**Figure 4.**
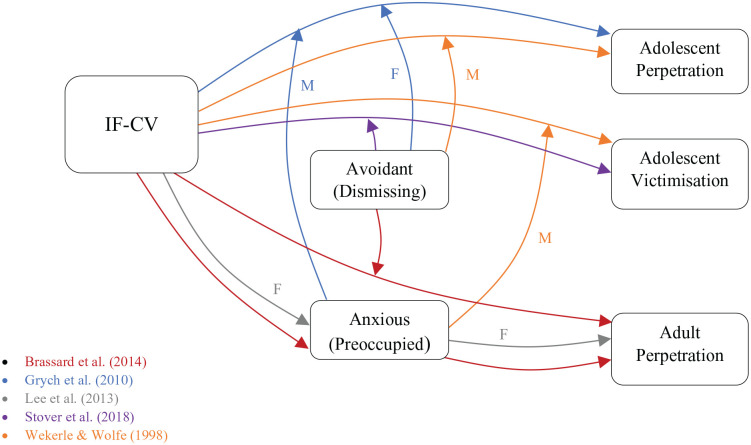
Significant mediating and moderating effects of attachment on the IF-CV to relationship violence pathway. *Note*. When there is an observed gender difference, results are shown using M = Male and F = Female. In Brassard et al. (2014), only males were recruited for the study.

A total of seven studies investigated the role of attachment styles (see Table 5). All of them are quantitative, using either mediation or moderation models to examine the pathway from IF-CV to relationship violence. Only one study (Brassard et al., 2014) tested both models simultaneously. The significant effects found are illustrated in Figure 4.

**Table 5.** Characteristics of Studies Examining Attachment as the Underlying Mechanism.

	Methods		Sample					
Main Author, Year	Quan	Qual	*N*	Sex	Age (M)	IF-CV	Type of Violence	Key Findings
Mediation								
Brassard, 2014	✓		302	302M	35.00	Child maltreatment (sexual)	Adult perpetration	• Attachment anxiety mediated the pathway from childhood sexual abuse to perpetrating violence in adult relationships.
Gay, 2013	✓		511	511F	19.14	Child maltreatment (emotional)	Adult violence	• No mediation found.
Kong, 2016	✓		479	NA	47.40	Child maltreatment	Adult victimization	• No mediation found.
Lee, 2013	✓		481	392F 89M	20.81	Child maltreatment	Adult perpetration	• No mediation found for men. • For women, attachment anxiety mediated the pathway from child maltreatment to perpetrating violence in adult relationships.
Moderation								
Brassard, 2014	✓		302	302M	35.00	Child maltreatment (sexual)	Adult perpetration	• Attachment avoidance moderated the pathway from childhood sexual abuse to perpetrating violence in adult relationships.
Grych, 2010	✓		391	204F 187M	15.60	Child maltreatment & witnessing domestic violence	Adolescent perpetration	• For boys, attachment anxiety moderated the relationship between IF-CV and adolescent relationship violence perpetration. • For girls, attachment avoidance moderated the relationship between IF-CV and adolescent violence perpetration.
Stover, 2018	✓		150	77M 73F	16.67	Child maltreatment	Adolescent violence	• Avoidant/dismissing attachment moderated the pathway from child maltreatment to being victimized in adolescent relationship violence. • Neither dismissing nor fearful attachment styles moderated the pathway from child maltreatment to perpetrating violence in adolescent relationships.
Wekerle, 1998	✓		321	128M 193F	15.13	Child maltreatment	Adolescent violence	For boys: • Avoidant attachment moderated the pathway from child maltreatment to perpetrating violence in adolescent relationships. • Anxious/ambivalent attachment moderated the pathway from child maltreatment to being victimized in adolescent relationship violence. For girls: • Although a heightened risk to perpetrate violence was observed among maltreated girls with insecure attachment, the predictive effect was not as substantial.

*Note*. IF-CV = intra-familial childhood victimization.

Out of the four mediational studies, two did not reveal any significant effects. Gay et al. (2013) found no evidence that attachment mediated the relationship between childhood emotional abuse and relationship violence. Similarly, Kong et al. (2018) reported no support for the pathway from child maltreatment to victimization. However, Lee et al. (2014) found that anxious attachment significantly mediated the effect of child maltreatment on perpetrating relationship violence in women but not in men. In contrast, Brassard et al. (2014) showed that anxious attachment significantly mediated the link between childhood sexual abuse and perpetration in their male-only sample.

On the other hand, all four moderational studies demonstrated significant effects of attachment on the pathway. Avoidant attachment significantly moderated the relationship between childhood sexual abuse and perpetration in adults (Brassard et al., 2014), as well as child maltreatment and victimization in adolescents (Stover et al., 2018). However, the effects may vary by gender as Grych and Kinsfogel (2010) found that the link between IF-CV and relationship violence was moderated by avoidant attachment in female adolescents and anxious attachment in male adolescents. This finding is supported by Wekerle and Wolfe (1998) who reported that anxiously attached male adolescents were more likely to be victimized, whereas those who were avoidantly attached were more likely to perpetrate. Although they observed a heightened risk for maltreated female adolescents with insecure attachment to engage in relationship violence perpetration, its predictive effect was not substantial.

### Early Maladaptive Schema

**Figure 5.**
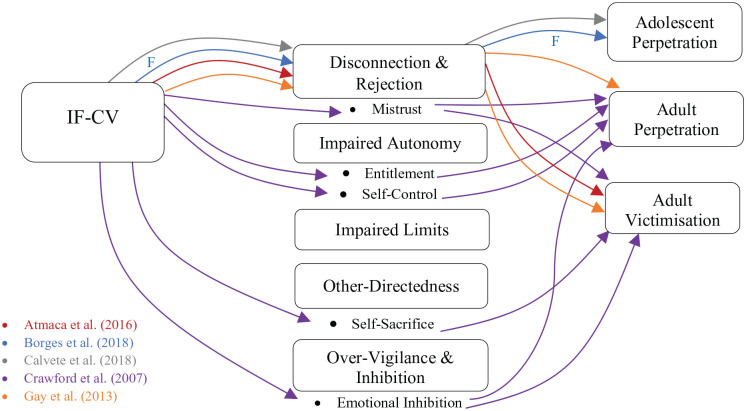
Significant mediating effects of EMS on the IF-CV to relationship violence pathway. *Note*. When there is an observed gender difference, results are shown using M = Male and F = Female. In Atmaca and Gençöz (2016), as well as Gay et al. (2013), only females were recruited for their studies.

A total of five quantitative studies examined the role of EMS in the pathway from IF-CV to relationship violence, using the mediational model (see Table 6 and Figure 5). Four of them found a significant mediational effect of the disconnection and rejection schema domain. For example, Borges and Dell’Aglio (2020) and Calvete et al. (2018) found that adolescents with a history of IF-CV were more likely to perpetrate relationship violence if they had developed the maladaptive beliefs of being disconnected and rejected. Atmaca and Gençöz (2016), as well as Gay et al. (2013), reported similar results in maltreated adult women who engaged in relationship violence either as perpetrators and/or victims.

**Table 6.** Characteristics of Studies Examining EMS as the Underlying Mechanism.

	Methods		Sample				Type of Violence	Key Findings
Main Author, Year	Quan	Qual	*N*	Sex	Age(M)	IF-CV		
Atmaca,2016	✓		222	222F	35.65	Child maltreatment	Adult victimization	• Disconnection and rejection domains mediated the pathway from child maltreatment to being victimized in adult relationship violence.
Borges, 2018	✓		397	156M 241F	14–19	Child maltreatment	Adolescent perpetration	• Disconnection and rejection domains mediated the pathway from child maltreatment to perpetrating relationship violence in adolescent girls but not boys.
Calvete, 2018	✓		867	409M 452F	13.77	Child maltreatment & witnessing domestic violence	Adolescent perpetration	• Disconnection and rejection domains mediated the pathway from IF-CV to perpetrating relationship violence in adolescence.
Crawford, 2007	✓		301	143M 158F	20.37	Child maltreatment (emotional)	Adult violence	• Schemas of mistrust and emotional inhibition mediated both relationships. • Schemas of self-control and entitlement mediated the pathway from childhood emotional abuse to perpetrating adult relationship violence. • Schema of self-sacrifice mediated the pathway from childhood emotional abuse to being victimized in adult relationship violence.
Gay, 2013	✓		511	511F	19.14	Child maltreatment (emotional)	Adult violence	• Only the disconnection and rejection domain mediated the relationship between childhood emotional abuse and adult relationship violence (perpetration and victimization).

*Note*. EMS = early maladaptive schema; IF-CV = intra-familial childhood victimization.

Crawford and Wright (2007) were the only researchers who unpacked the domains and investigated the roles of each EMS. They found that the schema of mistrust from the disconnection and rejection domain, and the schema of emotional inhibition from the over-vigilance and inhibition domain, significantly mediated the link between childhood emotional abuse and relationship violence in adulthood (perpetration and victimization). The schemas of entitlement and self-control from the impaired autonomy domain only mediated the relationship between childhood emotional abuse and perpetration. In contrast, the schema of self-sacrifice from the other-directedness domain only mediated the relationship between childhood emotional abuse and victimization.

### Sense-of-Self

**Figure 6.**
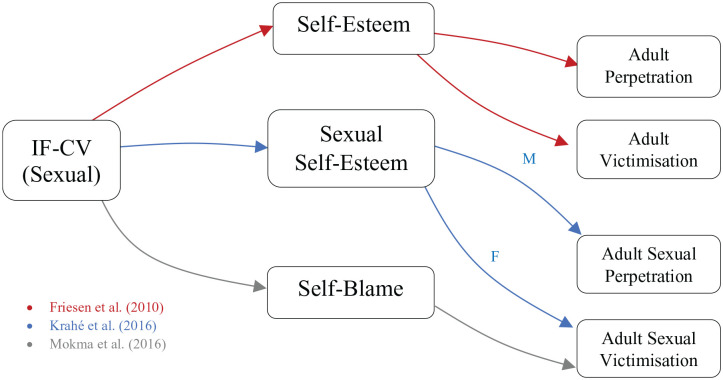
Significant mediating effects of sense-of-self on the IF-CV to relationship violence pathway. *Note*. When there is an observed gender difference, results are shown using M = Male and F = Female. In Mokma et al. (2016), only females were recruited for their study.

A total of six studies examined the role of the sense-of-self (see Table 7). Four of them were quantitative, with three using the mediational model to test the pathway from childhood sexual abuse to relationship violence (see Figure 6). In a 30-year longitudinal study, Friesen et al. (2010) found that self-esteem significantly mediated the relationship between childhood sexual abuse and relationship violence (perpetration and victimization). Krahé et al. (2016) specifically investigated the effect of sexual self-esteem and reported gender differences in their finding, that is, sexual self-esteem was a significant mediator for perpetration in males but victimization in women. Similarly, Mokma et al. (2016) found that self-blame mediated the pathway from childhood sexual abuse to sexual victimization in their female-only sample. In adolescents, O’Keefe (1998) reported a significant mediational effect of self-esteem on the link between witnessing domestic violence and perpetration, but only in males.

**Table 7.** Characteristics of Studies Examining Sense-of-Self as the Underlying Mechanism.

	Methods		Sample						
Main Author, Year	Quan	Qual	*N*	Sex	Age (M/Range)	IF-CV	Type of Violence	Underlying Factor	Key Findings
Friesen, 2010	✓		987	478M 509F	Birth-30	Child maltreatment (Sexual)	Adult violence	Self-esteem	• Self-esteem partially mediated the relationship between child sexual abuse and adult relationship violence.
Hoskins, 2020		✓	101	101M	35.18	IF-CV	Adult perpetration	Self-worth; self-blame; powerlessness	• Male perpetrators reported a history of IF-CV. This negatively impacted their self-worth and led to self-blame and a sense of powerlessness.
Keiski, 2017		✓	19	19F	21-63	IF-CV	Adult perpetration	Self-worth; self-blame; shame & guilt; powerlessness	• Female perpetrators reported being maltreated as a child. This had negatively impacted their self-worth and led to feelings of shame, guilt, and powerlessness.
Krahé, 2016	✓		2251	920M 1331F	21.3	Child maltreatment (sexual)	Adult violence (sexual)	Sexual self-esteem	• In men who were sexually abused as a child, sexual self-esteem mediated the relationship between child sexual abuse and perpetrating adult relationship violence. • In women who were sexually abused as a child, sexual self-esteem mediated the relationship between child sexual abuse and being victimized in adult relationship violence.
Mokma, 2016	✓		929	929F	18.89	Child maltreatment (sexual)	Adult victimization (sexual)	Self-blame	• Global self-blame mediated the relationship between child sexual abuse and sexual victimization in women.
O’Keefe, 1998	✓		232	94M 138F	16.90	Witnessing domestic violence	Adolescent violence	Self-esteem	• Low self-esteem differentiated between boys who perpetrated relationship violence and those who did not. • This effect was not found in girls.

*Note*. EMS = early maladaptive schema; IF-CV = intra-familial childhood victimization.

In comparison, only two studies used qualitative methods to investigate the mechanism. Hoskins and Kunkel (2020) examined the Sense-of-Self in male perpetrators who have experienced IF-CV. These men reported a pattern of low self-worth, high self-blame, and the feeling of powerlessness. Similarly, Keiski et al. (2018) found that female perpetrators with a history of IF-CV tended to express feelings of low self-worth, high self-blame, shame, guilt, and powerlessness because of the experiences.

## Discussion

By integrating the three influential developmental theories, namely Bowlby’s (1978) attachment styles, Young’s et al. (2003) EMS, and Erikson’s (1950) psychosocial development (i.e., self-development), researchers may be able to gain a deeper understanding of how IF-CV survivors appear to be at higher risks of engaging in relationship violence (Forke et al., 2018; Li et al., 2020). More effective interventions can be tailored as a result.

This systematic review aims to consolidate the empirical evidence in support of this theoretical conceptualization. As summarized in the result tables, the 17 studies included vary greatly in terms of the psychosocial factors of interest, and how the authors investigated their roles in the mechanism. For example, seven studies quantitatively tested the effect of attachment styles on the link between IF-CV and relationship violence. However, four of them used mediation analysis, while the other three used moderation analysis. While the mediational findings appear inconclusive (Brassard et al., 2014; Gay et al., 2013; Kong et al., 2018; Lee et al., 2014), results from all moderational studies indicated significant effects of attachment styles (Brassard et al., 2014; Grych & Kinsfogel, 2010; Stover et al., 2018; Wekerle & Wolfe, 1998), suggesting that they may function as a protective factor against the negative impact of IF-CV on relationship violence. In other words, among individuals who have experienced IF-CV, only those who are insecurely attached may perpetrate and/or become victimized later in life. As secure attachment appears to protect IF-CV survivors from repeating the pattern of violence, interventions targeting the changing of attachment styles in adolescence and adulthood may be particularly beneficial in preventing relationship violence among those at risk. In fact, early interventions to address attachment in childhood (e.g., family systems therapy) may serve as a crucial preventive practice to disrupt the onset of this cycle of violence.

**Figure 7.**
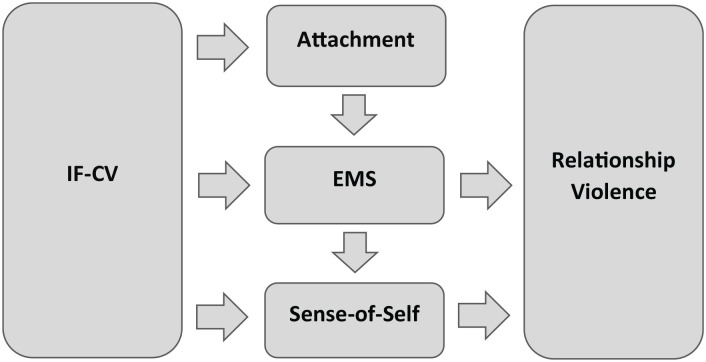
Revised model of the psychosocial mechanism underlying the pathway from IF-CV to relationship violence. *Note*. Please note that this figure provides a simplified depiction of the mechanisms involved. The authors acknowledge that it does not necessarily reflect the nuance and more complicated, dynamic, nature of the reality of trauma and violence.

These findings are partially in line with our conceptual model, as it has been theorized that early attachment patterns would influence later social interactions through the development of biased cognitive processing (negative IWMs or EMS; Riggs, 2010; see Figure 7 for the revised model). Consistently, all five studies, which examined the role of EMS in the mechanism, have yielded significant mediational results. Four of them found that the experience of IF-CV led to relationship violence through the adoption of the schema domain of Disconnection and Rejection (Atmaca & Gençöz, 2016; Borges & Dell’Aglio, 2020; Calvete et al., 2018; Gay et al., 2013). It is likely that being maltreated by caregivers would lead one to believe that they are being rejected by others. When this bias is carried over into intimate relationships, individuals may perceive conflicts as signs of rejection and react maladaptively by perpetrating or accepting violence.

In addition, Crawford and Wright (2007) unpacked the EMS domains and found the schema of mistrust from the Disconnection and Rejection domain to be the only significant mediator of the IF-CV to relationship violence pathway. This is in line with Erikson’s (1950) theorized crisis of trust versus mistrust in infancy. Trust also forms the basis of secure attachment according to Bowlby (1978). This finding therefore lends further support to our conceptual model that IF-CV may disrupt the early process of attachment forming, resulting in the development of an unhelpful thought pattern (i.e., the mistrust schema), which may further disrupt the process of forming an integrated Sense-of-Self and eventually contribute to relationship violence. However, none of the studies included directly examined the interactions between attachment, EMS, and Sense-of-Self. Hence, conclusive remarks can yet be drawn.

It is also worth noting that the process of forming trust and attachment extends beyond infancy. Therefore, IF-CV occurring during later childhood stages has the potential to disrupt trust and influence the formation of this EMS. In fact, past studies found that chronic and recent adversity appears to have the strongest impacts on life outcomes, as compared to early exposure (Flaherty et al., 2013; Thompson et al., 2015). Unfortunately, none of the papers in this review investigated the effect of IF-CV timing. Future research will benefit from taking this factor into consideration.

Furthermore, Crawford and Wright (2007) found significant mediational effects of schemas from other domains. For example, the schema of emotional inhibition from the over-vigilance and inhibition domain mediated the association between child maltreatment and the perpetration and victimization of relationship violence. This schema is characterized by the belief that one must excessively suppress one’s emotions. Hence, this result may highlight the other important role of the attachment system in intimate relationships, namely emotion regulation. Researchers have identified the inability to adaptively regulate emotions in times of conflict to be a risk factor for relationship violence among insecurely attached individuals (Spencer et al., 2021). Whether such a process may operate through a specific EMS will need to be further explored.

Lastly, the schemas of self-control and entitlement from the Impaired Limits domain, and the schema of self-sacrifice from the other-directedness domain, significantly mediated the relationships between child maltreatment and relationship violence perpetration and victimization, respectively (Crawford & Wright, 2007). These findings suggest that perpetrators may display deficits in self-control and may hold maladaptive beliefs that they are entitled to have their needs met at the expense of others. On the other hand, victims may hold the opposite belief that they have to abandon their own needs to serve others. It can be argued that these schemas reflect one’s view of self in the context of others—similar to the concept of interpersonal identity proposed by Erikson (1950). The inability to draw healthy distinctions between oneself and others has been theorized to precede relationship violence (Exner-Cortens, 2014). Although none of the studies included have investigated EMS in relation to identity formation, two qualitative research have described the disrupted Sense-of-Self to be prominent in IF-CV survivors who perpetrated (Hoskins et al., 2020; Keiski et al., 2017). They have identified the sense of powerlessness, low self-worth, and high self-blame to be pervasive among the samples. A quantitative study by Mokma et al. (2016) also demonstrated a significant mediational effect of self-blame on the link between childhood sexual abuse and sexual re-victimization in women. These findings provide preliminary support to the conceptual model that IF-CV may disrupt the process of identity formation through reinforcements of cognitive biases (i.e., EMS). Relationship violence may be perpetrated and/or accepted as part of an attempt to preserve the fragile Sense-of-Self.

Moreover, the included studies have revealed self-esteem to be another aspect of the Sense-of-Self crucial in the pathway. For example, Friesen et al. (2010) found self-esteem to be a significant mediator of childhood sexual abuse and relationship violence (perpetration and victimization). Krahé et al. (2016) specifically examined sexual self-esteem in childhood sexual abuse survivors and found that low sexual self-esteem led men to perpetrate, but women to be re-victimized. O’Keefe (1998) reported a similar gender effect on general self-esteem. They found that adolescent boys who witnessed violence at home were more likely to perpetrate if they had low self-esteem. However, the same effect was not observed in adolescent girls. These findings suggest the importance of targeting self-esteem in interventions to prevent IF-CV survivors from repeating the cycle of violence. They also highlight the need to further investigate how the pathway may vary for each gender.

In fact, gender differences are evident in other studies included in this review. For example, Grych and Kinsfogel (2010) found that anxious attachment increased the risk for adolescent boys with a history of IF-CV to engage in relationship violence, but avoidant attachment heightened this risk for adolescent girls. Wekerle and Wolfe (1998) also reported avoidant and anxious attachment to moderate the pathway from child maltreatment to male adolescent perpetration and victimization, respectively. Although a similar trend was observed among their female sample, the predictive effect was not substantial. Moreover, while Borges and Dell’Aglio (2020) found the Disconnection and rejection EMS domain to mediate the pathway from child maltreatment to perpetration in female adolescents only, other EMS studies did not indicate any gender effects. Findings are therefore inconclusive. In addition, seven studies recruited a single-gender sample, while one did not specify the demographic details of their participants. This limitation poses a challenge to further examine the nature of gender difference which may be at play in this mechanism. Future research may benefit from further exploring the interactions between gender, attachment styles, EMS, and disrupted Sense-of-Self among IF-CV survivors to better understand their unique risks of engaging in relationship violence.

## Conclusion

The 17 studies included in this review lend support to our conceptual model that IF-CV may disrupt the attachment system, leading to the formation of EMS and unresolved Sense-of-Self, which may contribute to the adoption of relationship violence in adolescence and adulthood. Attachment styles, in particular, have been identified as a potential protective factor (i.e., moderator) of the pathway. This yields significant implications for practitioners to target the attachment system when doing preventive work with individuals at risk. The insights into specific EMS prevalent among insecurely attached IF-CV survivors (e.g., mistrust) also allow practitioners to target unhelpful belief systems that may be associated with relationship violence in this population.

Furthermore, aspects of the Sense-of-Self, such as self-esteem, should be addressed to increase the sense-of-self worth and promote the overall sense of identity. Nevertheless, none of the studies included have examined attachment styles, EMS, and the Sense-of-Self in relation to one another. Moreover, identity resolution has not been investigated in this context of the cycle of violence. Although facets of the self-system have been identified as mediators of the pathway, it is ultimately the ability to define oneself in the relationship independently of one’s partner that has been theorized as crucial for the development of healthy intimacy (Erikson, 1950).

This review also pertains to other limitations. Since the authorship team was English-speaking, only studies conducted in English were included. It is therefore possible that relevant research conducted in other languages might have been omitted. In addition, all studies have defined relationship violence in the context of heteronormative intimacy, with the effect of gender being inconclusive. Most of the samples were also recruited from Western societies. The generalisability of the findings is therefore limited. It is crucial to acknowledge that the concept of identity resolution may vary across cultures, with collectivistic societies more likely to blur the line between self and intimate others.

Moreover, although the conceptual model highlights the importance of developmental stages, the effect of age during which IF-CV occurred has not been investigated in any of the studies. Past evidence has shown that chronic and recent adversity appear to have stronger impacts on life outcomes, as compared to early exposure (Flaherty et al., 2013; Thompson et al., 2015), suggesting the role of resilience across childhood development. Adolescence has also been identified as an especially vulnerable stage when both identity formation and the exploration of intimacy simultaneously take place. Since the ages of adolescents recruited vary greatly across the reviewed studies, their findings may inadvertently reflect the effects of developmental stages as younger adolescents may exhibit less resilience than their older peers. Future studies should address these gaps to better inform interventions suitable for different life stages.

Lastly, this systematic review synthesized research on childhood adversity under the operational definition of IF-CV. Out of the 17 studies included, 10 broadly defined their factor as child maltreatment and/or witnessing domestic violence at home, whereas four and three specifically investigated sexual and emotional abuse, respectively. It is important to note that there may be specific pathways pertaining to different types of adversity experienced. However, such differential pathways are not within the scope of this review, as we aim to present a broad model that could shed some light onto the complicated mechanism of trauma and violence. Future research may benefit from building on this model and investigating further the specific relationships between different types of adversity and later outcomes.

**Table:** Summary of Critical Findings.

Psychosocial Factor	Findings
Attachment	• Mediational studies have yielded inconsistent results. • Findings suggested insecure attachments significantly moderated the pathway from IF-CV to relationship violence.
EMS	• The Disconnection and Rejection domain emerged as a strong mediator of the pathway from IF-CV to relationship violence.
Sense-of-Self	• Self-control, self-blame, self-worth, and self-esteem emerged as important factors contributing to the pathway from IF-CV to relationship violence. • Sexual self-esteem, in particular, appeared to consistently mediate the relationship between experience of child sexual abuse and sexual abuse perpetration or victimization in adulthood.

**Table:** Implications for Practice, Theory, and Research.

	Implications
Theory Practice Research	• Attachment may function as a primary protective factor in the pathway from IF-CV to relationship violence, through its effects on EMS and Sense-of-Self. • Interventions targeting insecure attachments and their associated EMS may be crucial in breaking the cycle of violence. Measures to enhance positive Sense-of-Self should also be implemented. • Future studies should examine the interactions between attachment styles, EMS, and identity resolution. The effects of gender, culture, and age should also be explored.
